# Supplementary material for: Identification of photoperiod-induced specific miRNAs in the adrenal glands of Sunite sheep (Ovis aries)
Source: Front Vet Sci. 2022 Jul 22;9:888207. doi: 10.3389/fvets.2022.888207 (PMC9354845; doi:10.3389/fvets.2022.888207)
Supplement: Supplementary file 1 [file Data_Sheet_1.ZIP › Supplementary file/miRDeep2 file.zip!/miRDeep2 file/Know_Structure/LP42A1/oar-mir-26b.pdf]

miRBase precursor : oar-mir-26b
Total read count : 192760
oar-miR-26b read count : 192568
remaining reads : 192

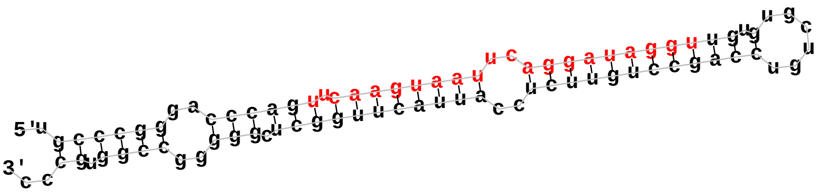

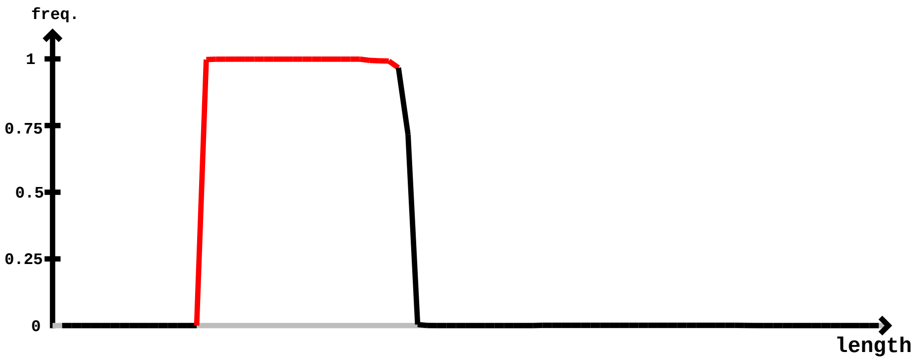


```
                 oar-miR-26b
5'- ugcccgggacccaguucaaguaauucaggauagguugugugcuguccagccuguucuccauuacuuggcucgggggccggugccc   -3'    exp
    .((((((..((((((.((((((((..(((((((((((.(......))))))))))))..))))))))))).))).)))).))..    reads      mm         sample
    .............guucaaguaauucaggauaggu..................................................    2          0          seq
    ..............uucaGguaauucaggau......................................................    2          1          seq
    ..............uucaaguaauucaggau......................................................    694        0          seq
    ..............uucaaguaauucaggUu......................................................    1          1          seq
    ..............uucaaguaaGucaggau......................................................    1          1          seq
    ..............uucaaUuaauucaggau......................................................    1          1          seq
    ..............uucaaguGauucaggau......................................................    3          1          seq
    ..............uCcaaguaauucaggau......................................................    3          1          seq
    ..............uucaUguaauucaggau......................................................    1          1          seq
    ..............Aucaaguaauucaggau......................................................    3          1          seq
    ..............uucaaguaauuUaggau......................................................    2          1          seq
    ..............uucaaguaauucaggaA......................................................    18         1          seq
    ..............uucCaguaauucaggau......................................................    49         1          seq
    ..............Gucaaguaauucaggau......................................................    1          1          seq
    ..............uuUaaguaauucaggau......................................................    2          1          seq
    ..............uucaaguaauucaggaG......................................................    23         1          seq
    ..............uucaaguaauucaggaC......................................................    84         1          seq
    ..............uucaaguaauucaggaAa.....................................................    2          1          seq
    ..............uucaaguaauucaggaCa.....................................................    3          1          seq
    ..............uucCaguaauucaggaua.....................................................    23         1          seq
    ..............uucaaguaauucaggaua.....................................................    298        0          seq
    ..............uucaaguaauucaggauag....................................................    163        0          seq
    ..............uucaaguaauucaggauagg...................................................    4820       0          seq
    ..............uucaaguaauucaggauaggu..................................................    48227      0          seq
    ..............uucaaguaauucaggauagguu.................................................    137129     0          seq
    ..............uucaaguaauucaggauagguug................................................    681        0          seq
    ..............uucaaguaauucaggauagguugu...............................................    62         0          seq
    ...............ucaaguaauucaggaua.....................................................    1          0          seq
    ...............ucaaguaauucaggauagg...................................................    7          0          seq
    ...............ucaaguaauucaggauaggu..................................................    46         0          seq
    ...............ucaaguaauucaggauagguu.................................................    166        0          seq
    ...............ucaaguaauucaggauagguug................................................    10         0          seq
    ...............ucaaguaauucaggauagguugu...............................................    1          0          seq
    ................caaguaauucaggauagg...................................................    1          0          seq
    ................caaguaauucaggauaggu..................................................    3          0          seq
```

oar-miR-26b

```
ugcccgggacccaguucaaguaauucaggauagguugugugcuguccagccuguucuccauuacuuggcucgggggccggugccc
................caaguaauucaggauagguu.................................................    23    0    seq
.................aaguaauucaggauagg...................................................    1     0    seq
.................aaguaauucaggauaggu..................................................    4     0    seq
.................aaguaauucaggauagguu.................................................    7     0    seq
.................................................ccuguucuccauuacuuggc................    7     0    seq
.................................................ccuguucuccauuacuuggcu...............    79    0    seq
.................................................ccuguucuccauuacuuggcuc..............    96    0    seq
.................................................ccuguucuccauuacuuggcucg.............    10    0    seq
```
